# A population-based study of transformed marginal zone lymphoma: identifying outcome-related characteristics

**DOI:** 10.1038/s41408-023-00903-w

**Published:** 2023-09-01

**Authors:** Johanna A. A. Bult, Francien Huisman, Yujie Zhong, Nick Veltmaat, Joost Kluiver, Sanne H. Tonino, Joost S. P. Vermaat, Martine E. D. Chamuleau, Arjan Diepstra, Anke van den Berg, Wouter J. Plattel, Mirian Brink, Marcel Nijland

**Affiliations:** 1 Department of Hematology, University Medical Center Groningen, Groningen, the Netherlands; 2 Department of Pathology and Medical Biology, University Medical Center Groningen, Groningen, the Netherlands; 3 Department of Hematology, Amsterdam University Medical Center, Amsterdam, the Netherlands; 4 Department of Hematology, Leiden University Medical Center, Leiden, the Netherlands; 5 Department of Research and Development, Netherlands Comprehensive Cancer Organization (IKNL), Utrecht, the Netherlands

**Keywords:** Cancer epidemiology, B-cell lymphoma

## Abstract

Histological transformation of marginal zone lymphoma (tMZL) into diffuse large B-cell lymphoma is associated with poor outcomes. Clinical characteristics associated with transformation risk and outcome after transformation are largely unknown due to scarcity of data. In this population-based study, competing risk analyses were performed to elucidate clinical characteristics associated with developing transformation among 1793 MZL patients using the Netherlands Cancer Registry. Cox regression analyses were performed to elucidate clinical characteristics associated with risk of relapse and mortality after transformation. Transformation occurred in 75 (4%) out of 1793 MZL patients. Elevated LDH and nodal MZL subtype at MZL diagnosis were associated with an increased risk, and radiotherapy with a reduced risk of developing tMZL. Most tMZL patients received R-(mini)CHOP (*n* = 53, 71%). Age >60 years and (immuno)chemotherapy before transformation were associated with an increased risk of relapse and mortality after transformation. Two-year progression-free survival (PFS) and overall survival (OS) were 66% (95% CI 52–77%) and 75% (95% CI 62–85%) for R-(mini)CHOP-treated tMZL patients, as compared to a PFS and OS both of 41% (95% CI 19–63%) for patients treated otherwise. Our study offers comprehensive insights into characteristics associated with transformation and survival after transformation, thereby optimizing guidelines and patient counseling.

## Introduction

Marginal zone lymphomas (MZLs) constitute 6–9% of mature B-cell neoplasms [1]. Annually, approximately 400 patients are diagnosed with MZL in the Netherlands [2]. The most common MZL subtypes are extranodal MZL (EMZL), nodal MZL (NMZL), and splenic MZL (SMZL) [1].

In general, MZLs are characterized by an indolent clinical course, with a 5-year relative survival estimate of 90% [3, 4]. Despite the favorable prognosis, approximately 4–18% of patients with MZL experience histological transformation into aggressive B-cell lymphomas, i.e. diffuse large B-cell lymphoma (DLBCL) [4–10]. Unlike newly diagnosed MZL, transformed MZLs (tMZLs) are associated with an aggressive clinical behavior and an inferior prognosis [4, 6, 10] with a 5-year overall survival (OS) of 65% [10].

Clinical characteristics at time of initial MZL diagnosis that have been associated with development of a transformation are: elevated levels of lactate dehydrogenase (LDH), advanced (III-IV) Ann Arbor stage, involvement of more than four nodal sites, a high follicular lymphoma International Prognostic Index (FLIPI) score, history of cancer, and failure to achieve complete remission after first-line treatment [8–10]. In addition, among patients with SMZL, presence of a complex karyotype at time of MZL diagnosis, defined by the presence of at least three clonal chromosomal aberrations, has been associated with an increased risk of developing a transformation [6].

Data on outcome after tMZL diagnosis are limited and prognostic factors associated with outcome among tMZL patients are largely unknown. As such, guidance on treatment decisions and counseling can be challenging for patients with tMZL. Failure to achieve a complete remission, advanced Ann Arbor stage, high-risk International Prognostic Index (IPI) score, and a tMZL <1 year [5, 6, 10] have been associated with an inferior survival after tMZL diagnosis. However, these findings are based on studies with limited numbers of patients, and not all studies encompassed all three MZL subtypes [5, 6]. Therefore, the aim of this study is to identify characteristics associated with outcome of tMZL in a nationwide, population-based study.

## Methods

### Netherlands Cancer registry and study population

The Netherlands Cancer Registry (NCR) [11] is a nationwide, population-based registry in which information on demographics and clinical characteristics is routinely collected by trained registrars through retrospective medical record review. Additional information on the NCR has been provided in the Supplementary.

Patients aged ≥18 years with a newly diagnosed MZL between January 1st, 2014 and December 31st, 2018 were identified in the NCR using the International Classification of Diseases for Oncology, 3rd edition (ICD-O-3) [12] of the World Health Organization (WHO) morphology code 9699/3 (EMZL and NMZL) and 9689/3 (SMZL). The MZL subtypes were distinguished in extranodal, nodal, and splenic subtype using first localization of presentation. Morphology code 9680/3 was used to identify histologically-proven transformations to DLBCL between January 1st, 2014 and December 31st, 2020. This time frame allowed a minimal follow-up of at least two years after MZL diagnosis to detect a transformation to DLBCL. A transformation was defined as a histologically-proven transformation from a MZL to DLBCL if the transformation occurred at least 3 months after MZL diagnosis, or if the transformation occurred within 3 months but after start of MZL treatment. Transformations that occurred within 3 months after untreated MZL diagnosis were excluded for this study.

### Risk factors

Clinical characteristics associated with developing a tMZL (to DLBCL), and relapse and mortality after tMZL diagnosis were evaluated. These characteristics included: age (categorized into ≤60 years and >60 years), gender, MZL subtype, Ann Arbor Stage (categorized into limited (stage I-II) and advanced (stage III-IV) Ann Arbor stage), elevated versus normal LDH, number of extranodal sites (categorized into <2 and ≥2 extranodal sites), WHO performance score (categorized into a score of <2 and ≥2), treatment group (categorized into no treatment (including surgery only and antibiotics only), radiotherapy, rituximab monotherapy, (immuno)chemotherapy, and other treatment), time to transformation (categorized into <2 years and ≥2 years), and (immuno)chemotherapy received for initial MZL (hereafter referred to as prior (immuno)chemotherapy).

### Endpoints

Endpoints included cumulative incidence of tMZL, overall survival post-diagnosis MZL (OS-1), overall survival post-diagnosis tMZL (OS-2), relative survival (RS) post-diagnosis tMZL, and progression-free survival post-diagnosis tMZL (PFS). Time to first event (transformation or death) was defined as the time between initial MZL diagnosis to date of first event. OS-1 was defined as time between initial MZL diagnosis and death by any cause, whereas OS-2 was defined as time between transformation and death by any cause. RS was defined as the ratio of the OS-2 to the expected OS of an equivalent group from the general population, matched by age and sex. As such, RS reflects the overall excess mortality associated with a tMZL diagnosis, thereby estimating disease specific survival in the absence of information on the cause of death. Progression-free survival (PFS) was defined as time between transformation and relapse of DLBCL or death by any cause, whichever came first.

### Statistical analysis

Distributions of age, gender, MZL subtype, Ann Arbor stage, and International Prognostics Index (IPI) [13] score at baseline of MZL diagnosis were compared between patients who did or did not develop a transformation. The Pearson χ2 test was used to compare categorical variables. The Kruskall–Wallis test was used to compare median age and median follow-up time. Kaplan-Meier estimates were used to analyze survival and the log-rank test was used to evaluate differences between survival curves.

Two analyses were conducted, labeled A and B. First, the cumulative incidence of tMZL and clinical characteristics associated with development of tMZL were evaluated in analysis A, using competing risk methods with death as competing risk. Due to immortal time bias inherent in the period until a transformation, a Cox regression analysis was performed, including transformation as time-varying covariate, in order to assess the association between transformation and mortality risk. All newly diagnosed MZL patients diagnosed between 2014 and 2018 were included with survival follow-up until December 31st, 2020, as transformations were monitored until this date. Secondly, survival rates after tMZL diagnosis and clinical characteristics associated with relapse and mortality after tMZL diagnosis were evaluated in analysis B, thereby including only tMZL patients who received treatment for their transformation. Of the tMZL patients, five did not receive treatment due to an unfit physical condition (*n* = 3) or to patient’s refusal (*n* = 2). These patients were excluded for further analyses. Survival follow-up was up to January 31st, 2023, resulting in at least two years post-diagnosis of tMZL for each patient.

Competing risk regression models were constructed with the Fine and Gray methodology [14] to identify clinical characteristics associated with developing a transformation, with death as competing risk. Using competing-risk analysis, sub-distribution hazard ratios (SHR) with 95% confidence intervals (CI) were estimated. The impact of the following variables at MZL diagnosis were assessed in the analysis for clinical characteristics associated with developing a tMZL (A): age, gender, MZL subtype, Ann Arbor stage, LDH, number of extranodal sites, WHO performance score, and treatment group.

Cox regression analyses were performed and hazard ratios (HR) with 95% CI were calculated to identify clinical characteristics associated with relapse and mortality after tMZL diagnosis. The impact of the variables used in analysis A at tMZL diagnosis were assessed in the analysis for clinical characteristics associated with relapse and mortality after tMZL diagnosis (B), except for treatment group. In addition, time to transformation and prior (immuno)chemotherapy were included as risk factors in analysis B.

For both competing risk regression analyses and Cox regression analyses, univariable analyses were performed and a multivariable model was built using a backwards stepwise regression process, whereby variables with a *p*-value < 0.05 were included in the final model.

*P*-values < 0.05 were considered statistically significant. All statistical analyses were performed with STATA version 17.0 (StataCorp, College Station, TX).

## Results

### Patient characteristics at MZL diagnosis

A total of 1793 patients with newly diagnosed MZL were identified in the NCR. Median age at diagnosis was 68 years (range 18–97), 51% were female, and 50% had a limited Ann Arbor stage (Table 1). According to localization of first appearance, 55% (*n* = 990) were diagnosed with EMZL, 30% (*n* = 537) with NMZL, and 15% (*n* = 266) with SMZL.

**Table 1:** Patient characteristics at time of marginal zone lymphoma diagnosis.

	All patients		Transformed		Non-transformed		*p*-value
	*n* = 1793		*n* = 75		*n* = 1718		
Female, no (%)	914	(51)	38	(51)	876	(51)	*0.96*
Age >60, no (%)	1256	(70)	50	(67)	1206	(70)	*0.51*
Age, median (range)	68	(18–97)	65	(18–87)	68	(18–97)	***<0.05***
MZL subtype, no (%)							***<0.001***
Extranodal	990	(55)	22	(29)	968	(56)	
Nodal	537	(30)	41	(55)	496	(29)	
Splenic	266	(15)	12	(16)	254	(15)	
Ann Arbor stage, no (%)							***<0.001***
I	697	(39)	12	(16)	685	(40)	
II	200	(11)	14	(19)	186	(11)	
III	130	(7)	15	(20)	115	(7)	
IV	715	(40)	34	(45)	681	(40)	
Unknown	51	(3)	0	(0)	51	(3)	
LDH, no (%)							***<0.001***
Normal	1335	(74)	47	(63)	1288	(75)	
Elevated	294	(16)	26	(35)	268	(16)	
Unknown	164	(9)	2	(3)	162	(9)	
Extranodal sites, no (%)							*0.38*
<2	1505	(84)	63	(84)	1442	(84)	
≥2	249	(14)	12	(16)	237	(14)	
Unknown	39	(2)	0	(0)	39	(2)	
WHO performance score, no (%)							*0.79*
<2	784	(44)	30	(40)	754	(44)	
≥2	51	(3)	2	(3)	49	(3)	
Unknown	958	(54)	43	(57)	915	(53)	
IPI-score, no (%)^a^							***<0.01***
≤2	1264	(71)	51	(68)	1213	(71)	
>2	313	(17)	22	(29)	291	(17)	
Unknown	216	(12)	2	(3)	214	(12)	
Treatment group, no (%)							
No treatment	917	(51)	40	(53)	877	(52)	***<0.01***
Radiotherapy only	384	(21)	4	(5)	380	(22)	
Rituximab monotherapy	70	(4)	3	(4)	67	(4)	
(Immuno)chemotherapy	402	(22)	27	(36)	375	(22)	
Other/unknown	20	(1)	1	(1)	19	(1)	

*MZL* marginal zone lymphoma, *LDH* lactate dehydrogenase, *IPI-score* International Prognostic Index score.

*Statistically significant *P*-vales (*P*-value <0.05) are presented in bold.

^a^Missing WHO performance scores were scored as 0 for the calculation of the IPI-score.

### Analysis A: clinical characteristics associated with transformation

**Fig. 1:**
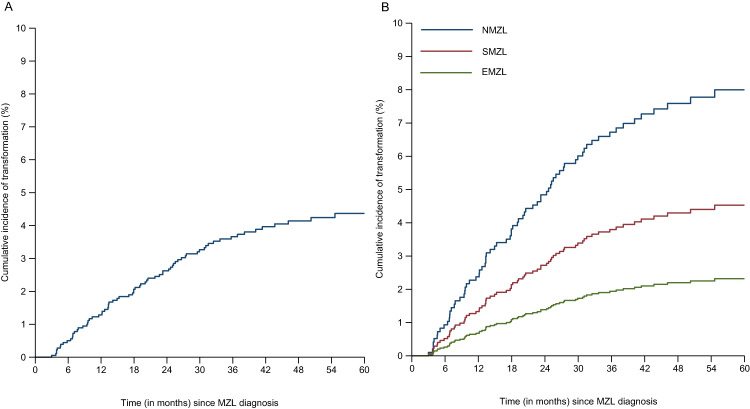
Cumulative incidence of transformation in 1 793 marginal zone lymphoma (MZL) patients between 2014 and 2020. **A** Cumulative incidence of transformation for the entire cohort of MZL patients; **B** Cumulative incidence of transformation per MZL subtype, NMZL nodal MZL, SMZL splenic MZL, EMZL extranodal MZL.

With a median follow-up time of 45 months (range 0-84) after initial MZL diagnosis, 4% (*n* = 75) of MZL cases developed a histologically-proven transformation to DLBCL. Median follow-up time for EMZL was 46 months (0–84), for NMZL 45 months (0-84), and for SMZL 42 months (1–84) (*p* < 0.05). Median time to transformation was 18 months (range 3–71). The 2-year cumulative incidence of transformation was 3% (95% CI 2–3%; Fig. 1A). The incidence rate of transformation was 11 per 1000 person-years (95% CI 9–14). No significant difference was observed in the incidence rate between the first year (13 per 1000 person-years) and second year (15 per 1000 person-years) of follow-up. A transformation was most frequently observed in NMZL (41 out of 537 (8%)), followed by SMZL (12 out of 266 (5%)), and EMZL (22 out of 990 (2%); *p* < 0.01) (Fig. 1B, Table 1). The majority of tMZL patients -- 47 out of 75 patients (63%) -- developed a tMZL within two years following MZL diagnosis (Table 2).

**Table 2:** Patient characteristics at time of transformed marginal zone lymphoma diagnosis.

	Total		R-(mini)CHOP		Other treatment		No treatment		p-value
	*n* = 75		*n* = 53		*n* = 17		*n* = 5		
Female, no (%)	38	(51)	29	(55)	8	(47)	1	(20)	*0.31*
Age >60, no (%)	54	(72)	37	(70)	12	(71)	5	(100)	*0.35*
Age, median (range)	67	(34–89)	66	(34–84)	67	(40–81)	77	(62–89)	*0.12*
MZL subtype, no (%)									*0.67*
Extranodal	22	(29)	17	(32)	5	(29)	0	(0)	
Nodal	41	(55)	28	(53)	9	(53)	4	(80)	
Splenic	12	(16)	8	(15)	3	(18)	1	(20)	
Ann Arbor stage, no (%)									*0.18*
Limited (I-II)	15	(20)	14	(26)	0	(0)	1	(20)	
Advanced (III-IV)	58	(77)	38	(72)	16	(94)	4	(80)	
Unknown	2	(3)	1	(2)	1	(6)	0	(0)	
LDH, no (%)									*0.54*
Normal	32	(43)	22	(42)	9	(53)	1	(20)	
Elevated	40	(53)	28	(53)	8	(47)	4	(80)	
Unknown	3	(4)	3	(6)	0	(0)	0	(0)	
Extranodal sites, no (%)									***0.03***
<2	53	(71)	38	(72)	11	(65)	4	(80)	
≥2	19	(25)	15	(28)	3	(18)	1	(20)	
Unknown	3	(4)	0	(0)	3	(18)	0	(0)	
WHO performance score, no (%)									*0.61*
<2	34	(45)	26	(49)	7	(41)	1	(20)	
≥2	2	(3)	1	(2)	1	(6)	0	(0)	
Unknown	39	(52)	26	(49)	9	(53)	4	(80)	
IPI-score, no (%)^a^									*0.53*
≤2	34	(45)	24	(45)	8	(47)	2	(40)	
>2	34	(45)	25	(47)	6	(35)	3	(60)	
Unknown	7	(9)	4	(8)	3	(18)	0	(0)	
Time to transformation <2 years, no (%)	47	(63)	34	(64)	10	(59)	3	(60)	*0.92*
Prior (immuno)chemotherapy, no (%)									***<0.001***
No	41	(55)	38	(72)	2	(12)	1	(20)	
Yes	33	(44)	14	(26)	15	(88)	4	(80)	
Unknown	1	(1)	1	(2)	0	(0)	0	(0)	

Statistically significant *P*-vales (*P*-value <0.05) are presented in bold.

*MZL* marginal zone lymphoma, *LDH* lactate dehydrogenase, *IPI-score* International Prognostic Index score.

*P-values are compared between the three treatment groups: R-(mini)CHOP (R-CHOP: *n* = 49, R-miniCHOP: *n* = 4), other treatment (including R-DHAP (*n* = 9), BR (*n* = 2), R-PECC (*n* = 2), R-CVP (*n* = 1), R-GDP (*n* = 1), CHOP21 (*n* = 1), and R-MBVP (*n* = 1)), and no treatment.

^a^Missing WHO performance scores were scored as 0 for the calculation of the IPI-score.

Risk factors associated with occurrence of tMZL from uni- and multivariable assessment are presented in Supplementary Table S1. In the multivariable analysis, elevated LDH (SHR 2.03, 95% CI 1.24–3.33) and a NMZL subtype (SHR 2.31, 95% CI 1.43–3.74) were associated with an increased risk of tMZL development. In addition, MZL patients treated with radiotherapy had a reduced risk of tMZL development compared to MZL patients who did not receive treatment (SHR 0.27, 95% CI 0.09–0.76).

**Fig. 2:**
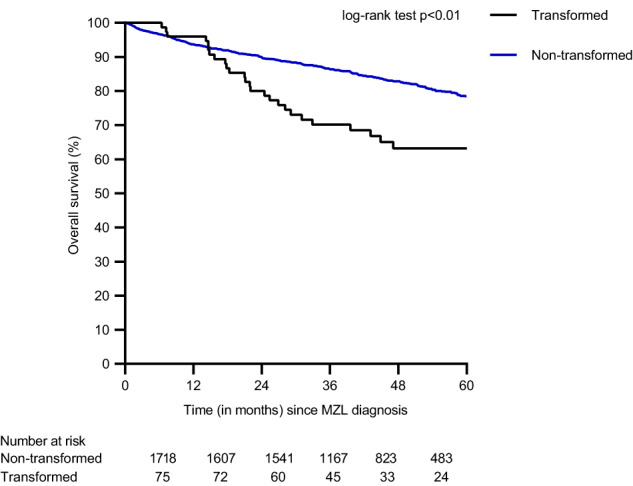
Overall survival (OS-1) of marginal zone lymphoma patients with or without a transformation. The black line indicates transformed MZL patients and the blue line indicates non-transformed MZL patients.

Five-year OS-1 estimates were 78% (95% CI 75–80%) (Supplementary Fig. S1) for all MZL patients, and 63% (95% CI 51–73%) and 78% (95% CI 76–81%) for patients with or without a tMZL, respectively (*p* < 0.01; Fig. 2). In the multivariable analysis with transformation as time-varying covariate, transformation (HR 2.72, 95% CI 1.82–4.06), as well as age >60 years (HR 4.51, 95% CI 3.13–6.51), NMZL subtype (HR 1.41, 95% CI 1.13–1.78), elevated LDH (HR 2.11, 95% CI 1.64–2.71), and WHO performance score ≥2 (HR 4.61, 95% CI 2.98–7.15) were associated with an increased risk of mortality. A female gender (HR 0.80, 95% CI 0.65–0.98) and radiotherapy compared to no treatment (HR 0.55, 95%CI 0.38-0.78) were associated with a reduced mortality risk (Supplementary Table S2).

*Analysis B: Clinical characteristics associated with outcome after transformation* Median age at tMZL diagnosis was 67 years (range 34–89), and 58 out of 75 (77%) had advanced stage disease at tMZL diagnosis (Table 2). In total, 33 (44%) tMZL patients received prior (immuno)chemotherapy and 41 (55%) did not. For one (1%) tMZL patient, treatment at initial MZL diagnosis was unknown. The median number of prior lines of (immuno)chemotherapy was 1 (1–3).

At time of transformation, most tMZL patients were treated with rituximab combined with cyclophosphamide, doxorubicin, vincristine, and prednisolone (R-CHOP) (*n* = 49 (65%)), or R-miniCHOP (*n* = 4 (8%)). Median age of patients treated with R-(mini)CHOP was 66 years (34–84). Among the remaining 22 (29%) tMZL patients, 11 (15%) were treated with salvage chemotherapy, consisting of rituximab combined with cisplatin, cytosine arabinoside and dexamethasone (R-DHAP) (*n* = 9), rituximab, gemcitabine, cisplatin, and dexamethasone (R-GDP) (*n* = 1), and rituximab, methotrexate, BCNU, VP16, and methylprednisolone (R-MBVP) (*n* = 1). Six patients (8%) were treated with less intensive treatment, consisting of rituximab, prednisolone, etoposide, chlorambucil, and lomustine (R-PECC) (*n* = 2), bendamustine and rituximab (BR) (*n* = 2), rituximab, cyclophosphamide, vincristine, and prednisolone (R-CVP) (*n* = 1), and CHOP only (*n* = 1). Five patients (7%) did not receive treatment. Most patients treated with salvage chemotherapy or less intensive treatment (*n* = 15 (88%)) received prior immunochemotherapy, including R-CHOP (*n* = 6) and R-CVP (*n* = 9). Patients treated with less intensive treatment were older (median age 72 years (59–81)) compared to patients with salvage chemotherapy (median age 64 years (40–72)) (*p* = 0.02)).

**Fig. 3:**
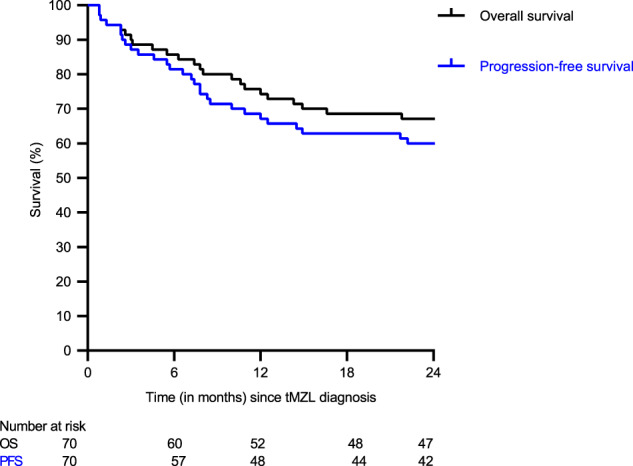
Progression-free survival and overall survival of transformed marginal zone lymphoma patients. The black line indicates the overall-survival after tMZL diagnosis, and the blue line indicates the progression-free survival after tMZL diagnosis.

**Fig. 4:**
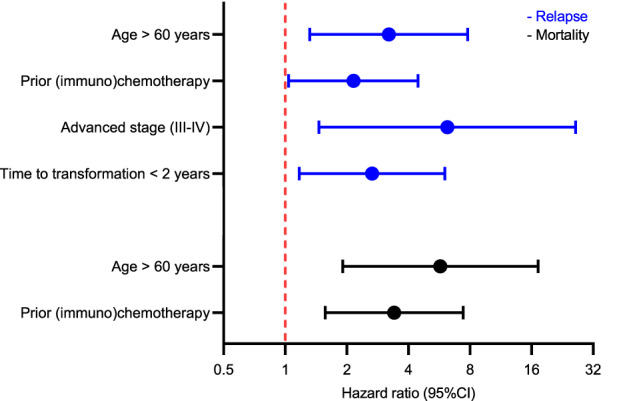
Forest plot of multivariable analysis for risk of relapse and mortality among transformed marginal zone lymphoma patients. Hazard ratios and 95% confidence intervals (CIs) are presented in blue for risk factors associated with relapse and in black for risk factors associated with mortality.

The median follow-up time after tMZL diagnosis for the 70 tMZL patients who received treatment was 41 months (range 1–97). The 2-year PFS and OS-2 rates were 60% (95% CI 48–70%) and 67% (95% CI 55–77%), respectively (Fig. 3). The 2-year RS was 69% (95% CI 56–79%). Nine (13%) tMZL patients relapsed within a median time of eight months (range 5–25) and in four (6%) tMZL patients, the disease progressed. In the multivariable analysis, age >60 years (HR 3.21, 95% CI 1.32–7.79), advanced stage (HR 6.20, 95% CI 1.46–26.27), time to transformation <2 years (HR 2.66, 95% CI 1.17–6.04), and prior (immuno)chemotherapy (HR 2.16, 95% CI 1.04–4.46) were associated with an increased risk of relapse (Fig. 4, Supplementary Table S3). Age >60 years (HR 5.73, 95% CI 1.91–17.23), and prior (immuno)chemotherapy (HR 3.41, 95% CI 1.57–7.42) were associated with a higher risk of mortality (Fig. 4, Supplementary Table S4).

The 2-year PFS, OS-2, and RS rates for tMZL patients treated with R-(mini)CHOP (*n* = 53) were 66% (95% CI 52–77%), 75% (95% CI 62–85%), and 78% (95% CI 63–87%), respectively. In comparison, for tMZL patients who received alternative treatment regimens (*n* = 17), the 2-year PFS and OS-2 rates were both 41% (95% CI 19–63%; Supplementary Fig. S2), and the 2-year RS was 42% (95% CI 19–64%).

## Discussion

In this population-based study among MZL patients, the 2-year cumulative incidence of transformation was 3%. Outcome of patients who developed a transformation was worse compared to MZL patients without a transformation. At MZL diagnosis, elevated LDH and a nodal MZL subtype were associated with an increased risk of developing a transformation. Age >60 years and prior (immuno)chemotherapy were identified as poor prognostic factors for survival after tMZL.

The low cumulative incidence of transformation as observed in the current study is in line with previous reports with 5-year cumulative incidence rates of 2.5–6.6% [4, 5, 7, 8, 10]. However, some studies have reported higher 5-year incidence rates, as much as 10% [6, 9]. Most transformations occurred within two years following MZL diagnosis. While some studies [10, 15, 16] have suggested that the incidence of histological transformation in indolent lymphomas may reach a plateau phase after ~15 years, the duration of follow-up in our current study is too short to comment on that. Consistent with previous reports, our study also showed that an elevated LDH at initial MZL diagnosis was associated with a higher risk of developing a transformation [8, 10]. In addition, among the three subtypes, NMZL patients had the highest risk of developing a transformation. Interestingly, the use of radiotherapy was associated with a reduced risk of transformation. This could be attributed to the curative potential of radiotherapy in limited stage disease [17].

Due to the paucity of data on outcome after tMZL, guidance on treatment decisions can be challenging. Previous studies [4–6, 10] reporting on outcome after tMZL diagnosis were based on limited and heterogenous cohorts. The largest cohort including all three MZL subtypes consisted of 55 tMZL patients [4]. However, this study did not provide information on clinical characteristics or treatment at time of tMZL diagnosis. Other studies included a lower number of tMZL patients (*n* = 34) [10], and/or included only one MZL subtype [5, 6]. To the best of our knowledge, this study represents the most extensive population-based analysis to date, offering comprehensive insights into the treatment and outcomes of tMZL, thereby optimizing clinical guidelines and patient counseling.

In accordance with previous studies [4, 6, 10], MZL patients who developed a transformation had a worse OS compared to patients without a transformation. The 2-year PFS and OS after tMZL for treated patients was 60% and 67%, respectively, which was most evident for patients who received R-CHOP with a 2-year PFS and OS of 66% and 75%, respectively. The PFS and OS are similar to the PFS and OS of newly diagnosed DLBCL patients [18, 19]. These findings align with previous observations in follicular lymphoma and chronic lymphocytic leukemia, wherein the outcome of previously untreated patients with a transformation is similar to de novo DLBCL [20, 21]. In our cohort, most tMZL patients who received non-anthracycline based chemotherapy, such as R-DHAP, BR or R-PECC, had received prior (immuno)chemotherapy, such as R-CVP and/or R-CHOP, and had an unfavorable outcome after tMZL diagnosis. (Immuno)chemotherapy is often administered as a first-line treatment to MZL patients with an advanced stage disease [17, 22]. These patients might have a more aggressive tumor biology resulting in a worse prognosis. These findings suggest that for patients without prior anthracycline based chemotherapy, R-CHOP appears to be the preferred regimen, while patients with prior anthracycline-based chemotherapy present a subgroup of patients that requires tailored treatment guidelines for better outcomes.

The mechanism underlying transformation in MZL is not known. Therefore, there is a need for further studies to elucidate the underlying mutational landscape of tMZL. This may aid in early identification of MZL patients with a high chance of transformation and formulating more appropriate treatment guidelines for these patients.

The main strength of this study is the use of a nationwide, population-based registry with data available on clinical characteristics and treatment at time of MZL and tMZL diagnosis. Limitations of this study mainly pertain to the lack of long-term follow-up of MZL patients, which could have prevented us from detecting transformations after four years. In addition, as a central pathology review was not possible due to the design of the study, there could be potential misclassification.

In conclusion, elevated LDH and nodal MZL subtype showed a higher risk of developing tMZL. Radiotherapy was associated with a reduced risk of developing tMZL compared to no treatment. tMZL patients treated with R-(mini)CHOP had a relatively favorable outcome, which was comparable to the outcome in de novo DLBCL. Age >60 years at tMZL diagnosis and prior (immuno)chemotherapy were identified as unfavorable prognostic factors after transformation. These findings can be used to optimize current patient management and clinical guidelines.

### Supplementary information

Supplementary

## Data Availability

The data that support the findings of this study are available via IKNL. These data are not publicly available, and restrictions apply to the availability of the data used for the current study. However, these data are available upon reasonable request and with permission of IKNL.
