# Supplementary material for: A population-based study of transformed marginal zone lymphoma: identifying outcome-related characteristics
Source: Blood Cancer J. 2023 Sep 1;13(1):130. doi: 10.1038/s41408-023-00903-w (PMC10474107; doi:10.1038/s41408-023-00903-w)
Supplement: Supplementary file 1 — Supplementary [file 41408_2023_903_MOESM1_ESM.docx]

**Supplementary**

**A Population-based Study of Transformed Marginal Zone Lymphoma: Identifying Outcome-related Characteristics**

Johanna A.A. Bult^1^, Francien Huisman^1^, Yujie Zhong^1,2^, Nick Veltmaat^1^, Joost Kluiver^2^, Sanne H. Tonino^3^, Joost S.P. Vermaat^4^, Martine E.D. Chamuleau^3^, Arjan Diepstra^2^, Anke van den Berg^2^, Wouter J. Plattel^1^, Mirian Brink^5^, Marcel Nijland^1^ [m.nijland@umcg.nl](mailto:m.nijland@umcg.nl)

1. Department of Hematology, University Medical Center Groningen, the Netherlands
2. Department of Pathology and Medical Biology, University Medical Center Groningen, the Netherlands
3. Department of Hematology, Amsterdam University Medical Center, the Netherlands
4. Department of Hematology, Leiden University Medical Center, the Netherlands
5. Department of Research and Development, Netherlands Comprehensive Cancer Organization (IKNL), Utrecht, the Netherlands

**Supplementary methods**

**The Netherlands Cancer Registry**

The Netherlands Cancer Registry (NCR) is a nationwide, population-based registry that covers ≥95% of all malignancies in the Netherlands. Netherlands Comprehensive Cancer Organization (IKNL) hosts the NCR. All newly diagnosed malignancies in the Netherlands are reported to the NCR via the Nationwide Network of Histopathology and Cytopathology, and the National Registry of Hospital Discharges (i.e. inpatient and outpatient discharges). Information on diagnosis, date of birth, gender, disease topography and morphology, and first-line therapy is routinely recorded in the NCR by trained registrars through retrospective medical records review. Topography and morphology are coded according to the International Classification of Diseases for Oncology (ICD-O). Information on the vital status for all patients is obtained through annual linkage with the Nationwide Population Registries Network that holds vital statistics on all residents in the Netherlands.

**Ethics**

According to the Central Committee on Research involving Human Subjects, the type of retrospective observational cohort study used in this study does not require approval from an ethics committee in the Netherlands. The Privacy Review Board of the NCR approved the use of anonymized data for this study.

**Supplementary Figures**

**Figure legends**

**Supplementary Figure S1. Overall survival (OS-1) of 1 793 newly diagnosed marginal zone lymphoma (MZL) patients in the Netherlands between 2014 and 2018**

**Supplementary Figure S2. The 2-year progression-free survival and overall survival (OS-2) in 70 histologically-proven transformed marginal zone lymphoma (tMZL) patients A** Progression-free survival after transformation of transformed marginal zone lymphoma (tMZL) patients treated with R-(mini)CHOP versus other treatments**; B** Overall survival after transformation of transformed marginal zone lymphoma (tMZL) patients treated with R-(mini)CHOP versus other treatments

**Supplementary Figure S1**

**Supplementary Figure S2**

A

B

**Supplementary table S1. Competing risk model for the association between clinical characteristics at time of marginal zone lymphoma (MZL) diagnosis and the risk of developing a transformation among MZL patients in the Netherlands**

|  | Univariable | | | | Multivariable | | | |
| --- | --- | --- | --- | --- | --- | --- | --- | --- |
|  | SHR | 95% CI | *P*-value*** | SHR | | 95% CI | *P*-value* |  |
| Age |  |  |  |  | |  |  |  |
| ≤60 years | *1* | *reference* |  |  | |  |  |  |
| >60 years | 0.85 | 0.53 – 1.38 | *0.51* |  | |  |  |  |
| Gender |  |  |  |  | |  |  |  |
| Male | *1* | *reference* |  |  | |  |  |  |
| Female | 1.00 | 0.64 – 1.58 | *0.98* |  | |  |  |  |
| Subtype |  |  |  |  | |  |  |  |
| EMZL/SMZL | *1* | *reference* |  | *1* | | *reference* |  |  |
| NMZL | 2.84 | 1.80 – 4.47 | ***<0.001*** | 2.31 | | 1.43 – 3.74 | **<0.01** |  |
| Ann Arbor stage |  |  |  |  | |  |  |  |
| Limited (I – II) | *1* | *reference* |  |  | |  |  |  |
| Advanced (III – IV) | 2.05 | 1.27 – 3.30 | ***<0.01*** |  | |  |  |  |
| Unknown | 1.27^e-06^ | 7.91^e-07^-204^e-06^ | ***<0.001*** |  | |  |  |  |
| LDH |  |  |  |  | |  |  |  |
| Normal | *1* | *reference* |  | *1* | | *reference* |  |  |
| Elevated | 2.65 | 1.64 – 4.27 | ***<0.001*** | 2.03 | | 1.24 – 3.33 | **<0.01** |  |
| Unknown | 0.34 | 0.08 – 1.41 | *0.14* | 0.40 | | 0.10 – 1.64 | 0.20 |  |
| Extranodal sites |  |  |  |  | |  |  |  |
| < 2 | *1* | *reference* |  |  | |  |  |  |
| ≥2 | 1.15 | 0.62 – 2.14 | *0.65* |  | |  |  |  |
| Unknown | 2.24^e-06^ | 1.50^e-06^ – 3.36^e-06^ | ***<0.001*** |  | |  |  |  |
| WHO performance score |  |  |  |  | |  |  |  |
| <2 | *1* | *reference* |  |  | |  |  |  |
| ≥2 | 1.07 | 0.25 – 4.57 | *0.92* |  | |  |  |  |
| Unknown | 1.18 | 0.74 – 1.88 | *0.49* |  | |  |  |  |
| Treatment group |  |  |  |  | |  |  |  |
| No treatment | *1* | *reference* |  | *1* | | *reference* |  |  |
| Radiotherapy | 0.23 | 0.08 – 0.65 | ***<0.01*** | 0.27 | | 0.09 – 0.76 | ***0.01*** |  |
| Rituximab monotherapy | 1.05 | 0.32 – 3.40 | *0.94* | 0.91 | | 0.28 – 2.90 | *0.87* |  |
| (Immuno)  chemotherapy | 1.53 | 0.94 – 2.50 | *0.09* | 1.06 | | 0.65 – 1.74 | *0.80* |  |
| Other/unknown | 1.05 | 0.15 – 7.45 | *0.97* | 1.31 | | 0.19 – 8.81 | *0.78* |  |

**P-vales are compared with the reference category. Statistically significant P-vales (P-value <0.05) are presented in bold.*

*Abbreviations: SHR: sub-distribution hazard ratio, CI: confidence interval, EMZL: extranodal marginal zone lymphoma, SMZL: splenic marginal zone lymphoma, NMZL: nodal marginal zone lymphoma, LDH: lactate dehydrogenase*

**Supplementary table S2. Cox regression model for the association between clinical characteristics at time of marginal zone lymphoma (MZL) diagnosis and the risk of mortality among MZL patients in the Netherlands with transformation as time-varying covariate**

|  | Univariable | | | Multivariable | | |
| --- | --- | --- | --- | --- | --- | --- |
|  | HR | 95% CI | *P*-value*** | HR | 95% CI | *P*-value* |
| Age |  |  |  |  |  |  |
| ≤60 years | *1* | *reference* |  | *1* | *reference* |  |
| >60 years | 4.98 | 3.46 – 7.16 | ***<0.001*** | 4.51 | 3.13 - 6.51 | ***<0.001*** |
| Gender |  |  |  |  |  |  |
| Male | *1* | reference |  | *1* | reference |  |
| Female | 0.91 | 0.74 – 1.12 | *0.37* | 0.80 | 0.65 - 0.98 | ***0.03*** |
| Subtype |  |  |  |  |  |  |
| EMZL/SMZL | *1* | *reference* |  | *1* | *reference* |  |
| NMZL | 1.41 | 1.14 – 1.75 | ***<0.01*** | 1.41 | 1.13 - 1.78 | **<0.01** |
| Ann Arbor stage |  |  |  |  |  |  |
| Limited (I – II) | *1* | *reference* |  |  |  |  |
| Advanced (III – IV) | 1.65 | 1.32 – 2.05 | ***<0.001*** |  |  |  |
| Unknown | 6.02 | 4.00 – 9.06 | ***<0.001*** |  |  |  |
| LDH |  |  |  |  |  |  |
| Normal | *1* | *reference* |  | *1* | *reference* |  |
| Elevated | 2.56 | 2.02 – 3.25 | ***<0.001*** | 2.11 | 1.64 - 2.71 | ***<0.001*** |
| Unknown | 1.70 | 1.22 – 2.37 | *<0.01* | 1.63 | 1.16 – 2.28 | ***<0.01*** |
| Extranodal sites |  |  |  |  |  |  |
| < 2 | *1* | *reference* |  |  |  |  |
| ≥2 | 1.07 | 0.79 – 1.44 | *0.67* |  |  |  |
| Unknown | 3.83 | 2.40 – 6.10 | ***<0.001*** |  |  |  |
| WHO performance score |  |  |  |  |  |  |
| <2 | *1* | *reference* |  | *1* | *reference* |  |
| ≥2 | 6.13 | 3.98 – 9.45 | ***<0.001*** | 4.61 | 2.98 - 7.15 | ***<0.001*** |
| Unknown | 2.01 | 1.59 – 2.54 | ***<0.001*** | 1.68 | 1.32 – 2.15 | ***<0.001*** |
| Treatment group |  |  |  |  |  |  |
| No treatment | *1* | *reference* |  | *1* | *reference* |  |
| Radiotherapy | 0.37 | 0.26 – 0.52 | ***<0.001*** | 0.55 | 0.38 - 0.78 | ***<0.01*** |
| Rituximab monotherapy | 0.92 | 0.53 – 1.61 | *0.77* | 0.80 | 0.45 – 1.42 | *0.44* |
| (Immuno)  chemotherapy | 0.99 | 0.77 – 1.26 | *0.92* | 0.89 | 0.69 – 1.16 | *0.39* |
| Other/unknown | 0.73 | 0.27 – 1.97 | *0.54* | 1.55 | 0.57 – 4.22 | *0.39* |
| Transformation |  |  |  |  |  |  |
| No | *1* | *reference* |  | *1* | *reference* |  |
| Yes | 2.95 | 1.99 – 4.37 | ***<0.001*** | 2.72 | 1.82 – 4.06 | ***<0.001*** |

**P-vales are compared with the reference category. Statistically significant P-vales (P-value <0.05) are presented in bold.*

*Abbreviations: HR: hazard ratio, CI: confidence interval, EMZL: extranodal marginal zone lymphoma, SMZL: splenic marginal zone lymphoma, NMZL: nodal marginal zone lymphoma, LDH: lactate dehydrogenase*

**Supplementary table S3. Cox regression model for the association between clinical characteristics at time of transformation and the risk of relapse among treated transformed marginal zone lymphoma patients in the Netherlands**

|  | **Univariable** | | | **Multivariable** | | |
| --- | --- | --- | --- | --- | --- | --- |
|  | HR | 95% CI | *P*-value*** | HR | 95% CI | *P*-value* |
| **Age** |  |  |  |  |  |  |
| ≤60 years | *1* | *reference* |  | *1* | *reference* |  |
| >60 years | 1.88 | 0.85 – 4.15 | *0.12* | 3.21 | 1.32 – 7.79 | ***0.01*** |
| **Gender** |  |  |  |  |  |  |
| Male | *1* | *reference* |  |  |  |  |
| Female | 1.20 | 0.62 – 2.36 | *0.59* |  |  |  |
| **Subtype** |  |  |  |  |  |  |
| EMZL/SMZL | *1* | *reference* |  |  |  |  |
| NMZL | 2.16 | 1.07 – 4.35 | ***0.03*** |  |  |  |
| **Ann Arbor stage** |  |  |  |  |  |  |
| Limited (I – II) | *1* | *reference* |  | *1* | *reference* |  |
| Advanced (III – IV) | 3.63 | 1.11 – 11.88 | ***0.03*** | 6.20 | 1.46 – 26.27 | ***0.01*** |
| Unknown | 4.00 | 0.41 – 39.74 | *0.23* | 24.03 | 1.83 – 316.24 | ***0.02*** |
| **LDH** |  |  |  |  |  |  |
| Normal | *1* | reference |  |  |  |  |
| Elevated | 2.32 | 1.12 – 4.81 | ***0.02*** |  |  |  |
| Unknown | 2.76 | 0.60 – 12.58 | *0.19* |  |  |  |
| **Extranodal sites** |  |  |  |  |  |  |
| < 2 | *1* | *reference* |  |  |  |  |
| ≥2 | 1.22 | 0.58 – 2.58 | *0.60* |  |  |  |
| Unknown | 2.70 | 0.63 – 11.56 | *0.18* |  |  |  |
| **WHO performance score** |  |  |  |  |  |  |
| <2 | *1* | *reference* |  |  |  |  |
| ≥2 | 1.20 | 0.16 – 9.14 | *0.86* |  |  |  |
| Unknown | 1.60 | 0.81 – 3.17 | *0.18* |  |  |  |
| **Prior (immuno)chemotherapy** |  |  |  |  |  |  |
| No | *1* | *reference* |  | *1* | *reference* |  |
| Yes | 2.02 | 1.02 – 4.00 | ***0.04*** | 2.16 | 1.04 – 4.46 | ***0.04*** |
| Unknown | 3.49 | 0.45 – 26.86 | *0.23* | 86.58 | 5.65 – 1327.34 | ***<0.01*** |
| **Time transformation** |  |  |  |  |  |  |
| ≥2 years | *1* | *reference* |  | *1* | *reference* |  |
| <2 years | 1.69 | 0.81 – 3.54 | 0.16 | 2.66 | 1.17 – 6.04 | ***0.02*** |

**P-vales are compared with the reference category. Statistically significant P-vales (P-value <0.05) are presented in bold.*

*Abbreviations: HR: hazard ratio, CI: confidence interval, EMZL: extranodal marginal zone lymphoma, SMZL: splenic marginal zone lymphoma, NMZL: nodal marginal zone lymphoma, LDH: lactate dehydrogenase*

**Supplementary table S4. Cox regression model for the association between clinical characteristics at time of transformation and the risk of mortality among treated transformed marginal zone lymphoma patients in the Netherlands**

|  | **Univariable** | | | **Multivariable** | | |
| --- | --- | --- | --- | --- | --- | --- |
|  | HR | 95% CI | *P*-value*** | HR | 95% CI | *P*-value* |
| **Age** |  |  |  |  |  |  |
| ≤60 years | *1* | *reference* |  | *1* | *reference* |  |
| >60 years | 3.34 | 1.28 – 8.73 | ***0.01*** | 5.73 | 1.91 – 17.23 | ***<0.01*** |
| **Gender** |  |  |  |  |  |  |
| Male | *1* | *reference* |  |  |  |  |
| Female | 1.35 | 0.67 – 2.73 | *0.41* |  |  |  |
| **Subtype** |  |  |  |  |  |  |
| EMZL/SMZL | *1* | *reference* |  |  |  |  |
| NMZL | 2.48 | 1.17 – 5.25 | ***0.02*** |  |  |  |
| **Ann Arbor stage** |  |  |  |  |  |  |
| Limited (I – II) | *1* | *reference* |  |  |  |  |
| Advanced (III – IV) | 12.21 | 1.65 – 90.33 | ***0.01*** |  |  |  |
| Unknown | 15.07 | 0.93 – 245.21 | *0.06* |  |  |  |
| **LDH** |  |  |  |  |  |  |
| Normal | *1* | *reference* |  |  |  |  |
| Elevated | 2.79 | 1.27 – 6.14 | ***0.01*** |  |  |  |
| Unknown | 4.02 | 0.85 – 19.02 | *0.08* |  |  |  |
| **Extranodal sites** |  |  |  |  |  |  |
| < 2 | *1* | *reference* |  |  |  |  |
| ≥2 | 1.23 | 0.56 – 2.70 | *0.60* |  |  |  |
| Unknown | 3.28 | 0.75 – 14.27 | *0.11* |  |  |  |
| **WHO performance score** |  |  |  |  |  |  |
| <2 | *1* | *reference* |  |  |  |  |
| ≥2 | 1.46 | 0.19 – 11.27 | 0.72 |  |  |  |
| Unknown | 1.47 | 0.72 – 3.01 | 0.29 |  |  |  |
| **Prior (immuno)chemotherapy** |  |  |  |  |  |  |
| No | *1* | *reference* |  | *1* | *reference* |  |
| Yes | 2.37 | 1.16 – 4.87 | ***0.02*** | 3.41 | 1.57 – 7.42 | ***<0.01*** |
| Unknown | 5.12 | 0.65 – 40.24 | *0.12* | 26.17 | 2.47 – 276.99 | ***<0.01*** |
| **Time transformation** |  |  |  |  |  |  |
| ≥2 years | *1* | *reference* |  |  |  |  |
| <2 years | 1.59 | 0.73 – 3.45 | *0.23* |  |  |  |

**P-vales are compared with the reference category. Statistically significant P-vales (P-value <0.05) are presented in bold.*

*Abbreviations: HR: hazard ratio, CI: confidence interval, EMZL: extranodal marginal zone lymphoma, SMZL: splenic marginal zone lymphoma, NMZL: nodal marginal zone lymphoma, LDH: lactate dehydrogenase*
